# 

**Published:** 1900-01-06

**Authors:** 


					222 THE HOSPITAL. Jan. 6, 1900.
NOTICE TO CORRESPONDENTS.
All MS., Letters, Boots for Keview, and other matters intended for
the Editor should bo addressed The Editor, "The Hospital," The
Hospital Building, 28 & 29, Southampton Street, Strand, W.O.
The Editor cannot undertake to return rejected MS., even when
accompanied by stamped directed envelope.
ITotico to Advertisers and Subscribers.
Ail Advertisement;!, Orders for Copies of The Hospital, and Business
Communications should ba addressed to MAXTACrZHt
(Kot to the Editor), The Hospital, The Hospital Building, 28 & 29,
Southampton Street, Strand, London, W.O.
The Cost of Subscription, post free, is as follows:?Per week, 2Jd.;
per quarter, 2s. 8d.; per half-year, 5s. 3d.; per annum, 10s. 6d. Foreign:?
1'cr annum, 15s. 2d., payable, in all cases, in advance.
SreiTICE.-Vol. X5VX. of " The Hospital" (April, 1309, to
September, 1889), in handsome cloth binding-,
is now on sale at the Office of this- Journal,
price 7s. Sd. Cases for binding- the I? umbers contained
in this Volume Is. 6d. Index to Vol. XXVI., 2?d., post
free.
Monthly Part for January is now ready. Price
Gd., by post 3d.
NOTES ON BOOKS.
Among the many diaries which have come to hand we
would draw special attention to the useful and time-
saving publications called "Every-Hour Diaries," published
by Messrs. Eason and Son, of Dublin. By carefully marking
off the space allotted to each day into appropriate divisions
for each of the working hours, method and punctuality are-
much encouraged. We can imagine nothing better, for a busy
professional man whose every day is crowded with appoint-
ments, and who constantly finds it desirable to see at a glance
what vacant time he has at his disposa,! than this arrangement.
BOOKS RECEIVED.
William Clay, Edinburgh.
" Practical Text-Book of Midwifery for Nurses and Students." By
Robert Jardine, M.D.
Macmillan and Co.
"A System of Medicine." Edited by Thomas Clifford Alibutt, M.A.,
M.D., LL.D., F.R.C.P., FfR.S. Volume VIII.
The Chemist and Druggist.
" The Chemists and Druggists' Diary, 1900."
George Bell and Sons.
" The Lute and Lays." By Charles Stuart Welles, M.D.
John Wright and Co.
"Pye's Surgical Handicraft." Fourth edition. Revised and edited
by Bertram M. H. Rogers, B.A., M.D.
Charles William Deacon.
" The Dream." A Phantasy. By George H. R. Dubbs, M.D.
Periodicals and Pamphlets.?Literary Digest?Scalpel?Q-ny's.
Hospital Gazette?London Hospital Gazette?Sunday at Home?Boy's
Own Paper?Open Court?Trained Nurse?Post Graduate?Asylum
News?Medical Press?Medical Record?Sanitary Record.
234 THE HOSPITAL. Ja*. 6, 1900.
Scraps and Gleanings.
The expenditure for the past year at the Salop Infirmary was ?5,893,
?whilst the receipts were ?5,400.
The expenditure during the past year of the Victorian Convalescent
Home for Surrey Women at Bognor was ?769, while the receipts for
income were ?804.
Last year the church collections in aid of the Glasgow Hospital Sunday
Fund amounted to ?3,951 odd, the amount contributed by the Sabbath
schools being ?427 odd.
The number of patients treated in the wards of the Dublin Children's
Hospital daring the past year was 454; the number treated in the
Extern Department was 3,328.
The new children's wards recently opened in connection with the
Brighton Workhouse contain accommodation for about 100 children.
The cost of erection has been just under ?15,000.
The ordinary income for the past year of the Stirling Royal Infirmary
is the largest in the history of the institution, viz., ?1,301 9s. 10d.; the
?ordinary expenditure amounted to ?1,179 13s. 9d.
A ftjether sum of ?20,000, in addition to the ?18,000 already in hand, is
required to build the new dispensary attached to the Glasgow Infirmary,
and to place the dispensary on a site most convenient for the public.
The number of out-patients treated during 1899 at the Boston Hos-
pital was 213 (including 64 out-patients), which is an increase of 15 over
1898. The total receipts showed a decrease of ?83, as compared
with 1898.
It is estimated that the house-to-house collection which the Mayor of
Swansea has suggested should be started on behalf of the Swansea Hos-
pital will bring in a clear profit to the hospital of between ?400 and ?500
per annum.
The new Victoria Hospital, Dundee, is now ready for the reception of
patients. It is capable of holding 32 patients, including one ward for
live cancer patients, and an isolation ward with two beds. The hospital
starts with 11 patients.
The first hospital in Dublin was founded in 1188 by a Dane, and was
situated in Thomas Street. It was dedicated to St. John the Baptist, and
in 1292 had been placed under the care of the St. Augustine Friars. In
1541, when all monasteries were suppressed in accordance with the Act
of Parliament, this hospital ceased to exist as a hospital and was used
for some time as a poorhouse fever hospital. It was demolished some
years ago.
The Tyrone County Cauneil liavo resolved t?< grant a, sum. of ?3,300 for
the building fund of the new county hospital, towards which ?6,000 has
been received, besides a special subscription of JS1.000 for the furnishing-
and completion of the internal arrangements.
The committee of the Forster Green Hospital Sor Consumption and
Diseases of the Chest, Belfast, has decided to take the very necessary step
of appointing a resident physician. This hospital is situated far from
the heart of the city and out of reach of the visiting staff except on the
prearranged visiting days, so that the appointment of a resident physician
will fill a much-felt want. It is hoped that the number of subscriptions
may be increased in order to provide the required funds for this post.
The Student of Medicine.
APPOINTMENTS.
E. P. Baujiann, M.B.Edin., M.R.C.S.Eng., appointed House
Surgeon to the Glasgow Maternity Hospital. W. F. C. Bennett,
L.R.C.P.E , L.R.C.S.E., appointed Assistant House Surgeon to
the Rotherliam Hospital and Dispensary. G. Fleming, M.B.,
C.M., M.R.C.P.E., appointed Medical Officer to the Ombersley
District of the Droitwich Union. J. H. Gough, F.R.C.S.Edin.,
L.R.C.P.Lond., appointed Honorary Physician to the Western
Hospital for Consumption, Torquay. R. H. Haves, M.R.C.S.,
appointed Assistant Ancesthetist to the Royal Ear Hospital. G.
Rose, M B., C.M., D.P.H.Aberd., appointed Surgeon to the
Royal Aberdeen Hospital for Sick Children. W. H. Spurgin,
M.R.C.S.Eng., appointed Medical Officer to the Western
Division of the Post Office, Newcastle.on-Tyne. A. C. Sturrock,
M.A., M.D.Edin., appointed Resident Medical Officer to the
Manchester Royal Infirmary. E. S. Wood, L.R.C.P., M.R.C.S.
appointed Medical Attendant at the Cambridge Borough Infec-
tious Diseases Hospital.
Royal London Ophthalmic Hospital, City Road, E.C.?The
following appointments have been made : Curator : W. T. Lister.
B.A., M.B., F.R.C.S. Senior House Surgeon: W. E. Smith,
M.B., C.M. Junior House Surgeon; A. F. MacCallan, B.A.,
M.B., B.C., F.R.C.S., L.R.C.P. Junior Out patient Surgical
Officer : F. A. C. Tyrrell, B.A., M.B., B.C., M R.C.S., L.R.C.P,
Jan. 6. 1900. THE HOSPITAL.
Hospitals, &c., Urgently Needing Annual Subscriptions Donations, and Legacies.
LONDON HOSPITAL, E.
The Committee appeal for ?40.000 a-year from voluntary contributions.
The number of IN-PATIENTS treated in 1897 was 11,146
? OUT-PATIENTS ? ? 161,033
Total number of Patients treated at the Hospital?172,179
Annual Subscribers are entitled to Tickets.
Thoroughly-Trained Private Nurses to be had immediately on application to the Matron.
Honble. SYDNEY HOLLAND, Chairman. G. Q. ROBERTS, House Governor.
THE UNIFORM SYSTEM OP ACCOUNTS,
AUDIT, AND TENDERS, for Hospitals and Institu-
tions, with Certain Checks upon Expenditure ; also the
Index of Classification. Compiled by a Committee of Hospital
Secretaries, and adopted by a General Meeting of the same, 18th
January, 1892. And certain Tender and other Forms for securing
Economy, By Sir HENRY BURDETT, K.O.B. Demy 8vo, pro-
fusely illustrated with Specimen Tables, Index of Classification,
Forms of Tender, &c., cloth extra, 6s.
ACCOUNT BOOKS FOE, INSTITUTIONS,
designed in acoordanoe with the Uniform System of
Accounts for Hospitals and Institutions. By Sir Henry
Btjkdett, K.O.B. Being a complete set of Account Books ruled in
accordance with the Uniform System adopted by the Metropolitan
Hospital Sunday Fund.
(Full Description of Books and Prices sent post free on application.)
London: The Scientific Press, Ltd., 28 & 29, Southampton Street,
Strand, W.O.
11 THE HOSPITAL " NURSING MIRROR. ^n.^isoo!"
"THE BURDETT SERIES"
OF POPULAR TEXT BOOKS ON NURSING.
Small Crown 8vo., clotht la. each.
No. 1.-PRACTICAL HINTS ON DISTRICT
NURSING By AMY HUGHES, late Superintendent
of the Metropolitan and National Nursing Association.
No. 2.?THE MATRON'S COURSE. An intro-
duction to Hospital and Private Nursing. By Miss S.
E. ORME.
No. 3.?THE MIDWIVES' POCKET-BOOK. By
HONNOR MORTEN. Author of " How to Become a
Nurse, and How to Succeed," &c., &c.
No. 4.?FEVERS AND INFECTIOUS DIS-
EASES : their Nursing and Practical Management.
By WILLIAM HARDING, M.D.Ed., M.R.C.P.Lond.,
Author of " Mental Nursing," &c.
No. 5.-NOTES ON FH ARM ACT AND DIS
FENSING FOB NURSES. By C. J. S.
THOMPSON.
No. 6.?THE RATIONAL USE OF ANTI-
SEPTICS IN MIDWIFERY FRACTICE.
By JAMES MORRISON, M.D., M.B., M.R.C.S.,
L.R.C.P.Lond. [In preparation.
No. 7?THE CARE OF CONSUMFTIVES. By
W. H. DAW, M.R.C.S., L.R.C.P.Lond.
No. 8 ?HOME NURSING OF SICK CHILD-
REN. By J. D. E. MORTIMER, M.B., F.R.C.S.,
L.S.A.
No. 9.?MENTAL NURSING}. By WILLIAM
HARDING, M.D.Ed., M.R.C.P.Lond.
London : The Scientific Press, Ltd., 28 & 29, Southampton Street, Strand, W.C.
TZBC?' "THE HOSPITAL" NURSING MIRROR.
NOW V**
A REPRINT OP
A Nursing Handbook
TOGETHER WITH S. MAW, SON, AND THOMPSON'S
COMPLETE CATALOGUE OF NURSING REQUISITES.
The above work is now republished at a cost of Is. per copy. Messrs. S. M.,
S., and T. have been compelled to make this charge on account of the
extreme popularity of the work and the great expense incurred in its production.
Post Free on receipt of Is-
\ S. JVIflW, S0& and TflOf/lPSON,
7 to 12, ALDERSGATE STREET, LONDON, E.C.
% <</

				

